# Case Report: immune checkpoint inhibitor-associated aseptic meningitis in lung cancer: two cases illustrating an exclusion-based diagnosis approach and stepwise immunosuppressive treatment

**DOI:** 10.3389/fonc.2026.1877315

**Published:** 2026-07-07

**Authors:** Hiroshi Nakahama, Shigeo Hanada, Eisuke Takeda, Yui Takahashi, Yuichiro Nei, Takahiro Mitsumura, Atsushi Miyamoto, Meiyo Tamaoka

**Affiliations:** 1 Department of Respiratory Medicine, Respiratory Center, Toranomon Hospital, Tokyo, Japan; 2 Okinaka Memorial Institute for Medical Research, Tokyo, Japan

**Keywords:** aseptic meningitis, cerebrospinal fluid, immune checkpoint inhibitor, interleukin-6, leptomeningeal carcinomatosis, lung adenocarcinoma, multiplex PCR, tocilizumab

## Abstract

**Background:**

Immune checkpoint inhibitor (ICI)-associated aseptic meningitis is uncommon neurological immune-related adverse event that can cause substantial morbidity if appropriate immunosuppressive therapy is delayed while excluding infectious meningitis or leptomeningeal carcinomatosis is being evaluated. We report two lung adenocarcinoma cases illustrating an exclusion-based diagnostic process and stepwise immunosuppressive treatment of suspected ICI-associated aseptic meningitis.

**Case description:**

Case 1: A 66-year-old woman experienced fever, neck stiffness, and altered mental status 13 days after initiating adjuvant atezolizumab. Cerebrospinal fluid (CSF) analysis showed pleocytosis and marked protein elevation, whereas neuroimaging remained unremarkable. After alternative diagnoses were excluded, high-dose intravenous methylprednisolone was administered, resulting in prompt improvement in consciousness and neurological symptoms, followed by oral corticosteroid tapering without recurrence. Case 2: A 75-year-old man receiving carboplatin/nab-paclitaxel with durvalumab plus tremelimumab experienced ICI-associated colitis followed by Common Terminology Criteria for Adverse Events grade 3 meningitis with impaired consciousness. CSF multiplex polymerase chain reaction, cytological analysis, and further investigations supported early consideration that common infections and leptomeningeal carcinomatosis were unlikely, enabling timely immunosuppression for suspected ICI-associated aseptic meningitis. However, neurological deterioration continued despite high-dose corticosteroids and intravenous immunoglobulin. Consequently, tocilizumab was administered as rescue therapy, following which, both clinical outcomes and CSF parameters improved.

**Conclusion:**

These two cases illustrated a practical clinical approach to suspected ICI-associated aseptic meningitis based on rapid exclusion of alternative diagnoses and stepwise immunosuppressive treatment. High-dose corticosteroids remain the cornerstone of initial therapy, whereas additional immunomodulatory treatment might be considered in select refractory cases. CSF multiplex polymerase chain reaction might serve as an adjunctive tool in some patients by helping exclude common treatable infections during early diagnostic evaluation.

## 1 Introduction

Immune checkpoint inhibitors (ICIs) have become integral to lung cancer management in perioperative and metastatic settings. However, in addition to their clinical benefits, ICIs can induce immune-related adverse events (irAEs), some of which require urgent consideration and treatment. The current guidelines promote early clinical recognition, exclusion of alternative etiologies, and severity-guided immunosuppression when an irAE is strongly suspected (1–3).

Neurological irAEs are less common than are dermatologic, endocrine, or gastrointestinal toxicities. However, irAEs are clinically relevant as delayed diagnosis and treatment can lead to substantial morbidity or even death. Among these events, ICI-associated aseptic meningitis presents a particular diagnostic challenge because its early symptomatic manifestations, including headache, fever, neck stiffness, and altered mental status, overlap with those of infectious meningitis and leptomeningeal carcinomatosis (4–7). Recent pharmacovigilance analyses have also identified a safety signal for ICI-associated aseptic meningitis, suggesting that although infrequently reported, it is typically serious and often occurs early after ICI initiation (8). However, clinicians might hesitate to initiate or intensify immunosuppressive therapy until possibilities of treatable infections and tumor-related meningeal disease have been reasonably excluded. This diagnostic uncertainty can delay appropriate treatment of ICI-associated aseptic meningitis.

Therefore, the key clinical issue is not simply the low frequency of this condition, but how to perform a timely exclusion-based diagnosis and initiate appropriate treatment without delay. Neuroimaging, cerebrospinal fluid (CSF) analysis, microbiological testing, and cytological evaluation contribute to this process, whereas a rapid CSF multiplex polymerase chain reaction (PCR) panel (FilmArray^®^ Meningitis/Encephalitis Panel) might serve as an adjunctive tool in selected patients by helping exclude common treatable infections early in the evaluation (9–11).

Herein, we report two cases of lung adenocarcinoma with ICI-associated aseptic meningitis that illustrate a practical clinical approach based on exclusion of alternative diagnoses and stepwise immunosuppressive treatment. The contribution of these cases lies in the structured exclusion-based diagnostic process and stepwise escalation of immunosuppressive therapy in a time-sensitive clinical setting. Across both cases, the major challenge was to distinguish ICI-associated aseptic meningitis from infection or leptomeningeal carcinomatosis without delay, while tailoring treatment escalation according to the clinical response.

## 2 Case description

### 2.1 Case 1

A 66-year-old woman with resected stage IIB lung adenocarcinoma harboring an epidermal growth factor receptor (EGFR) exon 19 deletion and programmed death-ligand 1 (PD-L1) tumor proportion score of 10% underwent video-assisted thoracoscopic left upper lobectomy followed by four courses of adjuvant cisplatin plus vinorelbine. Her Eastern Cooperative Oncology Group performance status was 0. Two months after completing adjuvant chemotherapy, adjuvant atezolizumab was initiated.

Thirteen days after the first dose of atezolizumab, the patient developed fever, neck pain, and fatigue. By day 18, behavioral changes and progressive dysarthria manifested, prompting an emergency admission. On presentation, her Glasgow Coma Scale (GCS) score was E3V2M5. Nuchal rigidity was present, whereas no focal neurological deficits were identified. Non-contrast brain computed tomography (CT) did not show any acute abnormalities. Laboratory tests (**Table 1**) demonstrated a modest inflammatory response (C-reactive protein, 2.92 mg/dL) without metabolic or endocrine abnormalities sufficient to explain the altered mental status. Furthermore, cerebrospinal fluid (CSF) examination revealed pleocytosis (74 cells/μL; monocytes, 53; neutrophils, 18; others, 3), marked protein elevation (577 mg/dL), and preserved glucose (77 mg/dL; serum glucose, 146 mg/dL). Since infectious meningitis, including bacterial, viral, and fungal etiologies, could not initially be excluded, empiric ceftriaxone, ampicillin, and acyclovir were started.

**Table 1:** Blood tests on admission, and cerebrospinal fluid (CSF) findings on day 18 in Case 1.

Blood tests on admission									
Category	Test	On admission	Unit	Reference	Category	Test	On admission	Unit	Reference
CBC	WBC	5.3	x10^3^/µL	3.3-8.1	Biochemistry and biomarkers	Amylase	**37**	U/L	44-132
	RBC	**3.53**	x10^6^/µL	4.35-5.55		Uric acid	**2.9**	mg/dL	3.7-7.0
	Hb	**10.3**	g/dL	13.7-16.8		BUN	23	mg/dL	8-20
	Ht	**31.3**	%	40.7-50.1		Cre	**0.62**	mg/dL	0.65-1.07
	MCV	88.7	fL	83.6-98.2		glucose	**144**	mg/dL	73-109
	Platelet	177	x10^3^/µL	158-348		Na	**137**	mmol/L	138-145
Endocrinology	TSH	**0.581**	µIU/mL	0.61-4.23		K	3.6	mmol/L	3.6-4.8
	Free T3	**2.03**	pg/mL	2.30-4.10		Cl	103	mmol/L	101-108
	Free T4	0.85	ng/dL	0.70-1.70		Ca	**8.4**	mg/dL	8.8-10.1
	ACTH	21.9	pg/mL	7.2-63.3		CRP	2.92	mg/dL	¥0.14
	Cortisol	**44.8**	µg/dL	7.1-19.6		CEA	2.5	ng/mL	≤5.0
	TP	**6.0**	g/dL	6.6-8.1		SLX	19.3	U/mL	≤38.0
	ALP	98	U/L	38-113		KL-6	166	U/mL	<500
Biochemistry and biomarkers	γ-GT	41	U/L	13-64		BNP	**362.4**	pg/mL	≦18.4

CSF findings				
Category	Test	On day 18	Unit	Reference
Cell count and differential	WBC	**74**	/µL	≦ 5
	Mononuclear	**53**	/µL	
	Polymorphonuclear	**18**	/µL	
	Others	3	/µL	
Chemistry	Protein	**577**	mg/dL	10-40
	Glucose	**77**	mg/dL	50-75
	Blood Glucose	**146**	mg/dL	73-109
Microbiology	HSV-1 DNA (PCR)	< 2×10^2^	copies/mL	< 2×10^2^
	HSV-2 DNA (PCR)	< 2×10^2^	copies/mL	< 2×10^2^
	Cryptococcus antigen	negative		negative

ACTH, adrenocorticotropic hormone; BNP, B-type natriuretic peptide; CBC, complete blood count; CEA, carcinoembryonic antigen; CLEIA, chemiluminescent enzyme immunoassay; CSF, cerebrospinal fluid; HSV, herpes simplex virus; KL-6, Krebs von den Lungen-6; PCR, polymerase chain reaction; SLX, sialyl Lewis X.

Bold values indicate abnormal findings.

On day 19, brain magnetic resonance imaging (MRI) remained unremarkable; no neurological improvement occurred. Given the temporal relationship with ICI initiation, the CSF profile and absence of radiographic findings suggestive of alternative central nervous system pathology, ICI-associated aseptic meningitis became a major diagnostic consideration. Concurrently, cryptococcal meningitis remained part of the differential diagnosis; moreover, amphotericin B plus flucytosine was initiated while further evaluation was ongoing. High-dose intravenous methylprednisolone (1 g/day for 3 days) was also started for suspected Common Terminology Criteria for Adverse Events (CTCAE) grade 3 ICI-associated aseptic meningitis.

**Figure 1:**
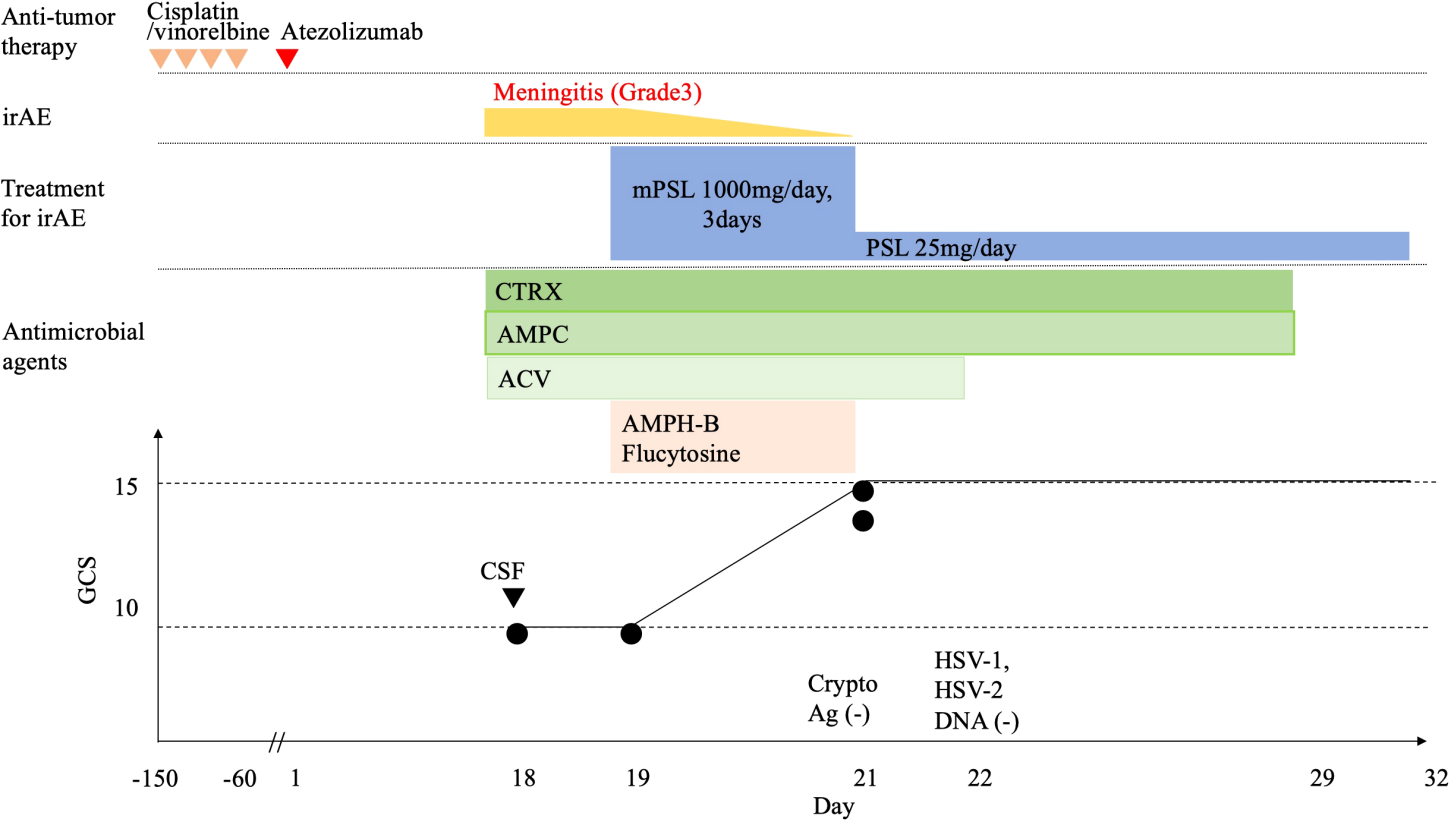
Clinical course, treatments, and Glasgow Coma Scale (GCS) scores in Case 1.The vertical axis indicates GCS scores, while the horizontal axis indicates days from atezolizumab initiation. ACV, acyclovir; AMPC, ampicillin; AMPH-B, amphotericin B; CTRX, ceftriaxone; flucytosine, 5-fluorocytosine; GCS, Glasgow Coma Scale; ICI, immune checkpoint inhibitor; irAE, immune-related adverse event; mPSL, methylprednisolone; PSL, prednisolone.

The patient’s consciousness and meningeal symptoms improved rapidly by day 21 (GCS, E4V5M6). Subsequent serum cryptococcal antigen testing, CSF cultures, and CSF PCR assays for herpes simplex virus and varicella-zoster virus showed negative results. Antifungal, antiviral, and antibacterial agents were, thereby, discontinued as microbiological data accumulated and the clinical course became more consistent with ICI-associated aseptic meningitis. Oral prednisolone at dose of 25 mg/day (approximately 0.5 mg/kg/day) was continued and gradually tapered. The patient was discharged on day 32 with full neurological recovery. No recurrence of meningitis or progression of lung cancer has been observed for more than 2 years of follow-up (**Figure 1**).

### 2.2 Case 2

A 75-year-old man underwent multiple lung resections of synchronous lung adenocarcinomas (pT1aN0M0, pT1bN0M0, and pT4N0M0; stage IIIA). The stage IIIA lesion harbored KRAS G12D and CTNNB1 mutations; the PD-L1 tumor proportion score was <1%. The patient’s Eastern Cooperative Oncology Group performance status was 0. Eight months postoperatively, chest CT demonstrated bilateral pulmonary metastases consistent with recurrent invasive mucinous adenocarcinoma. In response, treatment with carboplatin plus nab-paclitaxel combined with durvalumab and tremelimumab was initiated. On day 85 after treatment initiation, the patient experienced watery diarrhea requiring hospitalization. Colonoscopy and histopathology supported grade 2 ICI-associated colitis according to the CTCAEs. Prednisolone was started at a dose of 30 mg/day (0.5 mg/kg/day) on day 87 and escalated to 60 mg/day on day 92 due to persistent symptoms. On day 93, the patient experienced fever, headache, and hypotension, for which empiric piperacillin–tazobactam was started. By day 102, his headache worsened, accompanied by neck stiffness and impaired consciousness (GCS E3V4M6).

Brain and spinal MRI showed no acute abnormalities, while electroencephalography revealed only mild diffuse slowing without epileptiform discharges. CSF examination (**Table 2**) demonstrated lymphocytic pleocytosis (40 cells/μL; monocytes, 40; neutrophils, 0), elevated protein (207 mg/dL), relatively preserved glucose (57 mg/dL; serum glucose, 175 mg/dL), an increased immunoglobulin G index (0.85), and positive oligoclonal bands. Since infection and leptomeningeal carcinomatosis were important competing diagnoses, the patient underwent a structured exclusion-based evaluation. A rapid CSF multiplex PCR panel showed negative results within 2 hours after lumbar puncture. In addition, CSF cytology showed class II without malignant cells, and India ink and viral PCR testing also showed negative results. Taken together with the neuroimaging findings and clinical course, these observations supported early consideration that common infectious etiologies and leptomeningeal carcinomatosis were unlikely, making CTCAE grade 3 ICI-associated aseptic meningitis the most likely diagnosis. Although mild encephalitic involvement could not be fully excluded, meningitis was considered the predominant clinical syndrome. High-dose intravenous methylprednisolone (1 g/day for 3 days) was initiated, followed by oral prednisolone at 60 mg/day (1.0 mg/kg/day).

**Table 2:** Cerebrospinal fluid (CSF) findings on days 102, 110, and 119, and FilmArray® meningitis/encephalitis panel results from CSF on day 102 in Case 2.

Category	Reference	Unit	Day 102	Day 110	Day 119
CSF findings					
WBC	≦ 5	/µL	**40**	**39**	**19**
Mononuclear		/µL	**40**	**39**	**19**
Polymorphonuclear		/µL	0	0	0
Protein	10-40	mg/dL	**207**	**224**	**109**
Albumin		mg/dL	100.6	107.7	44.7
Glucose	50-75	mg/dL	**57**	**112**	**80**
IgG	2-4	mg/dL	**32.1**	**36.6**	**26.7**
IgG index	≦ 0.73		**0.85**	**0.41**	**0.73**
Blood Glucose	73-109	mg/dL	**175**	**325**	**248**
Oligoclonal bands	Negative		**Positive**	**Positive**	**Negative**
Myelin basic protein	≦ 102	ng/L	**158.1**	**225.7**	**147.5**
HSV-1 DNA (PCR)	< 2×10^2^	copies/mL	< 2×10²	–	–
HSV-2 DNA (PCR)	< 2×10^2^	copies/mL	< 2×10²	–	–
IL-6	Not established†	pg/mL	–	**75.1**	–
FilmArray^®^ ME panel					
·Bacteria					
*Escherichia coli K1*	not detected		not detected		
*Haemophilus influenzae*	not detected		not detected		
*Listeria monocytogenes*	not detected		not detected		
*Neisseria meningitidis*	not detected		not detected		
*Streptococcus agalactiae*	not detected		not detected		
*Streptococcus pneumoniae*	not detected		not detected		
·Viruses					
*Cytomegalovirus*	not detected		not detected		
*Enterovirus*	not detected		not detected		
*Herpes simplex virus 1*	not detected		not detected		
*Herpes simplex virus 2*	not detected		not detected		
*Human herpesvirus 6*	not detected		not detected		
*Human parechovirus*	not detected		not detected		
*Varicella zoster virus*	not detected		not detected		
· Yeast					
*Cryptococcus neoformans/gattii*			not detected		

CSF, cerebrospinal fluid; Meningitis/Encephalitis panel, ME Panel; HSV, herpes simplex virus; IL-6, interleukin-6.

† No institutional reference range is available for CSF IL-6; results were interpreted in the clinical context.

Bold values indicate abnormal findings.

**Figure 2:**
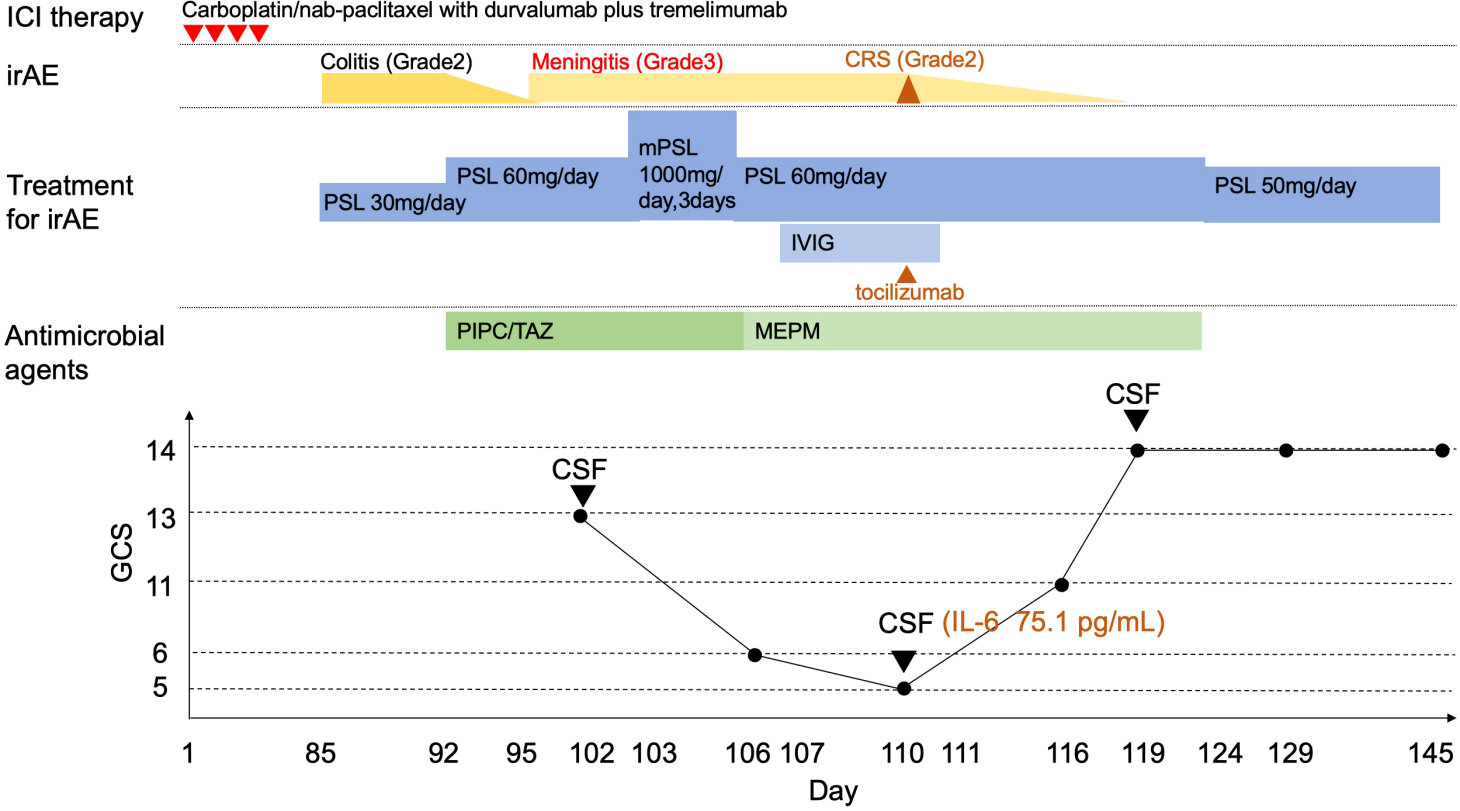
Clinical course, treatments, and Glasgow Coma Scale (GCS) scores in Case 2. The vertical axis indicates GCS scores, while the horizontal axis indicates days from initiation of the first ICI-containing regimen. Chemotherapy with ICI comprised carboplatin plus nab-paclitaxel combined with durvalumab and tremelimumab. CRS, cytokine release syndrome; GCS, Glasgow Coma Scale; ICI, immune checkpoint inhibitor; irAE, immune-related adverse event; IVIG, intravenous immunoglobulin; MEPM, meropenem; mPSL, methylprednisolone; PIPC/TAZ, piperacillin/tazobactam; PSL, prednisolone; TCZ, tocilizumab.

Despite the treatment, the patient’s neurological status deteriorated on day 106 (GCS E2V2M2). Head CT showed no hemorrhage or large infarction. Lacosamide was started because acute symptomatic seizures could not be completely excluded; however, repeated electroencephalography revealed no epileptiform activity or non-convulsive status epilepticus. Given bacterial meningitis had not yet been outrightly excluded, antimicrobial therapy was broadened to meropenem until culture results became available. Intravenous immunoglobulin (20 g/day for 5 consecutive days; total 100 g) on day 107 was administered, without clear neurological improvement. Repeat contrast-enhanced brain MRI on day 110 remained unremarkable. Repeat lumbar puncture showed persistent lymphocytic pleocytosis and elevated protein, with a normal opening pressure (95 mmH2O). Notably, CSF interleukin-6 (IL-6) was markedly elevated (75.1 pg/mL), whereas serum IL-6 was within the normal range (3.8 pg/mL). At that point, CSF cultures also revealed negative results. The elevated CSF IL-6 levels suggested intrathecal inflammation; however, the finding was not considered a stand-alone indication for tocilizumab. Given the continued neurological deterioration despite corticosteroids and intravenous immunoglobulin, together with persistent fever, fluid-responsive hypotension, concomitant cytokine release syndrome (CRS) grade 2 was suspected; therefore, tocilizumab 480 mg (8 mg/kg) was administered as a rescue therapy. Thereafter, his neurological status gradually improved (GCS E2V4M5 on day 116 and E4V4M6 on day 119), along with improvement in CSF findings. Prednisolone was gradually tapered. Meropenem was discontinued on day 124, after blood and CSF cultures were finalized as negative and the patient had clinically stabilized. He was discharged on day 145 with recovery of consciousness (**Figure 2**).

Diagnostic assessment: Diagnostic evaluation in both cases relied on integration of clinical presentation, cerebrospinal fluid findings, neuroimaging, microbiological testing, and cytological assessment, prioritizing exclusion of infectious meningitis and leptomeningeal carcinomatosis.

Therapeutic intervention and follow-up: Therapeutic interventions included high-dose corticosteroids in both cases, with additional immunomodulatory therapy in the second case. Clinical follow-up was based on neurological status and serial cerebrospinal fluid assessments, demonstrating resolution or improvement of symptoms.

## 3 Discussion

**Figure 3:**
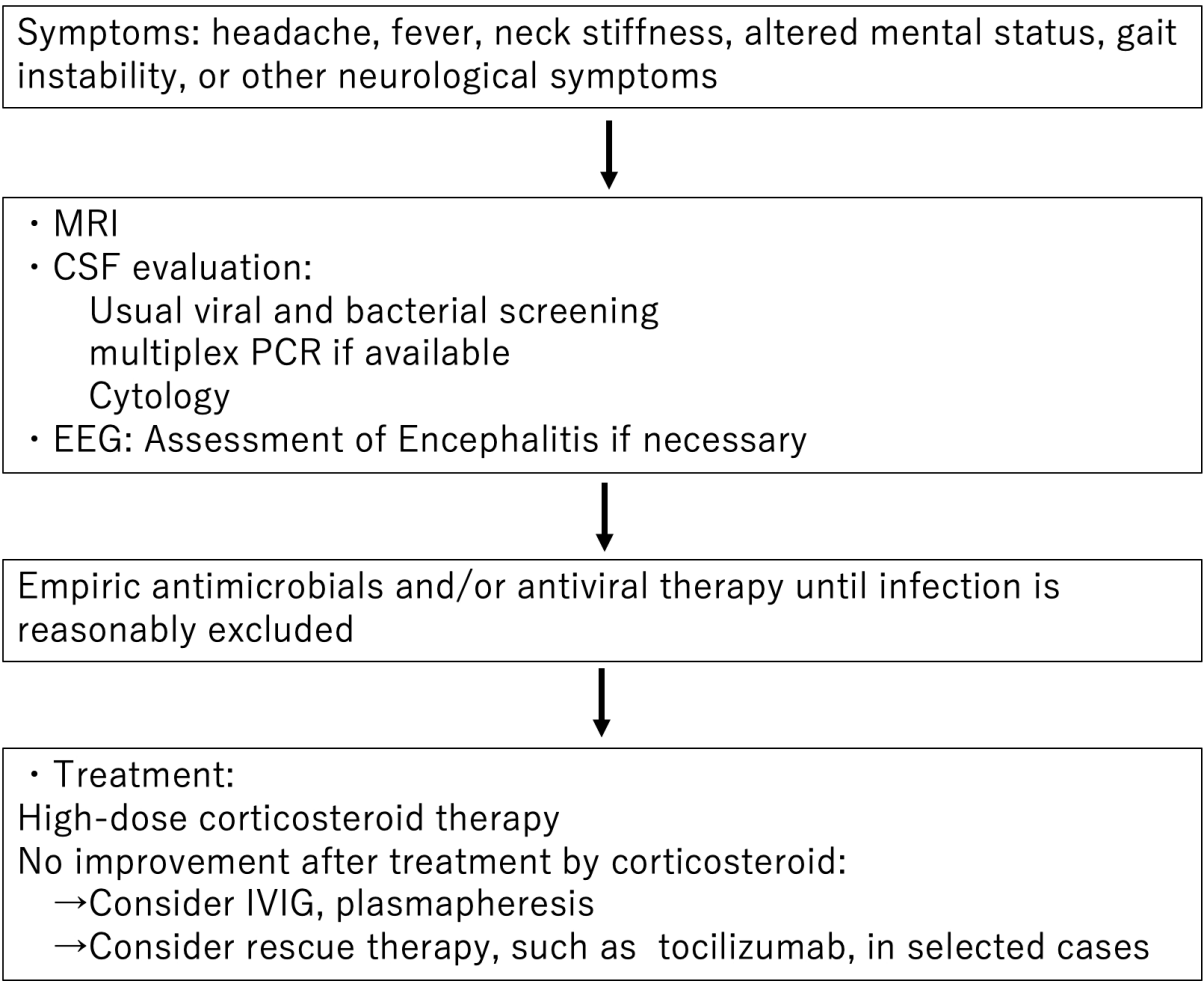
Proposed practical diagnostic and therapeutic approach for suspected ICI-associated aseptic meningitis. CSF, Cerebrospinal fluid; IVIG, intravenous immunoglobulin; MRI, magnetic resonance imaging; PCR, polymerase chain reaction.

ICI-associated aseptic meningitis is clinically important because diagnostic and therapeutic decisions are often time-sensitive. Although neurological toxicities associated with ICIs are infrequent, they require structured evaluation because their presentation may overlap with infection, vascular events, metabolic encephalopathy, seizure-related disorders, or tumor-associated neurological complications (4, 5). The present two cases illustrate a practical clinical approach to suspected ICI-associated aseptic meningitis centered on two principles: rapid exclusion of alternative diagnoses and stepwise immunosuppressive treatment based on clinical severity and response. This approach is illustrated in **Figure 3**.

The timing of the onset of ICI-associated aseptic meningitis varies. In a systematic review, the median number of treatment cycles at symptom onset was 2 cycles (range: 1–14) (6). In a recent pharmacovigilance study, the median time to onset was 34 days (range: 0–2,194) (8). This variability is reflected in our cases: Symptoms appeared 13 days after the first dose of atezolizumab in Case 1 and 93 days after the initiation of ICI-containing therapy in Case 2. Therefore, clinicians should maintain a high level of suspicion for ICI-associated aseptic meningitis in cases of neurological symptoms, regardless of whether onset is early or late, once alternative diagnoses have been appropriately ruled out.

A major diagnostic challenge is the overlap in symptoms with infectious meningitis and leptomeningeal carcinomatosis. In patients with cancer who present with fever, headache, neck stiffness, or altered mental status during ICI therapy, these competing diagnoses must be considered urgently. In the presented cases, diagnostic reasoning relied on integration of clinical presentation, CSF findings, neuroimaging, microbiological studies, and cytological assessment. In the second case, the rapid CSF multiplex PCR panel contributed to the early exclusion of common treatable infections; however, it should be viewed as only a part of the combined evaluation rather than a stand-alone diagnostic test. A negative result from the panel does not exclude all infectious etiologies and must be interpreted together with routine CSF parameters, cultures, imaging, cytology, and the subsequent clinical course (9–11). Thus, the value of the panel in this setting is adjunctive: It might support earlier exclusion-based decision-making in select patients, but it does not replace comprehensive diagnostic assessment.

Empirical broad-spectrum antimicrobial therapy might be reasonable when bacterial meningitis has not been excluded, even if ICI-associated aseptic meningitis is suspected. This is particularly crucial when neurological symptoms worsen (1). In Case 2, meropenem was administered because bacterial meningitis remained a concern prior to initiating further immunosuppression. However, prolonged broad-spectrum antimicrobial use could increase the risk of antimicrobial resistance, Clostridioides difficile infection, and drug-related adverse events. Antimicrobial therapy should be reassessed and narrowed or discontinued once cultures, the rapid CSF multiplex PCR panel, imaging, and the clinical course make bacterial infection unlikely, in accordance with antimicrobial stewardship principles (12).

Once infection and leptomeningeal carcinomatosis are deemed unlikely and the overall findings support a diagnosis of ICI-associated aseptic meningitis, high-dose corticosteroid therapy remains the cornerstone of treatment (1–7). Our first case followed this expected pattern, demonstrating prompt neurological improvement after methylprednisolone. In contrast, the second case demonstrated that some patients may not stabilize with corticosteroids alone. Prior literature on corticosteroid-refractory irAEs suggests that a subset of immune toxicities might persist, recur, or require treatment escalation despite standard steroid therapy (13). From a practical perspective, these cases support a stepwise management framework in which corticosteroids remain the first-line treatment, whereas additional immunomodulatory therapy might be considered in select refractory cases.

In this context, tocilizumab should be cautiously interpreted as a rescue option rather than a standard treatment of ICI-associated aseptic meningitis. Evidence supporting IL-6 receptor blockade for irAEs remains limited; however, systematic reviews and case-series data suggest possible benefit in select steroid-refractory situations after appropriate exclusion of infection (14). Reports of elevated CSF IL-6 levels in ICI-induced autoimmune meningoencephalitis also provide a biological rationale for considering IL-6-targeted therapy in some patients with persistent hyperinflammatory central nervous system manifestations (15, 16). No validated reference range for CSF IL-6 has been established in patients with ICI-associated aseptic meningitis. However, the CSF IL-6 level in Case 2 was well above the 10 pg/mL cutoff reported for central nervous system inflammatory diseases (17). Importantly, an elevated CSF IL-6 should not be regarded as an established indication for tocilizumab in this setting. In our second case, tocilizumab was introduced after continued neurological deterioration despite high-dose corticosteroids and intravenous immunoglobulin, in the context of suspected concomitant grade 2 CRS, persistent inflammatory findings, and markedly elevated CSF IL-6 levels. This experience should be regarded as hypothesis-generating rather than practice-defining; however, it suggests that IL-6 receptor blockade might be considered in carefully select refractory cases.

This report has some limitations. First, the implications of rapid CSF multiplex PCR panel and tocilizumab in this report were each based on a single case; therefore, their generalizability is severely limited. Second, causality cannot be definitively proven in either case, as exclusion-based diagnosis inherently leaves slight residual uncertainty. Third, concomitant irAEs, prior antimicrobial exposure, and systemic inflammatory conditions might have influenced both the clinical course and interpretation of treatment response. Fourth, the panel does not exclude all infectious etiologies and should not be overinterpreted. Finally, evidence supporting IL-6 blockade in ICI-associated meningitis remains sparse, and larger multicenter registries or prospective studies are necessary for better defining its role.

In conclusion, these two cases highlight a practical clinical approach to suspected ICI-associated aseptic meningitis based on the exclusion of alternative diagnoses and stepwise immunosuppressive treatment. Since the clinical presentation could overlap with infectious meningitis and leptomeningeal carcinomatosis, a prompt multimodal evaluation is essential. Once ICI-associated aseptic meningitis is considered highly likely, early high-dose corticosteroid therapy remains the mainstay of treatment. A rapid CSF multiplex PCR panel might serve as an adjunctive tool in select patients by helping exclude common treatable infections during early evaluation, whereas tocilizumab might be considered only as a rescue therapy in carefully select refractory cases.

## 4 Patient perspective

### 4.1 Patient perspective (Case 1; family): “Our family was alarmed by the sudden neurological symptoms and feared infection or cancer progression. We appreciated the prompt evaluation, clear explanations, and rapid improvement after treatment.”

### 4.2 Patient perspective (Case 2; family): “We were distressed by the rapid neurological decline and uncertainty about the cause. We valued the timely evaluation, clear communication, and improvement after stepwise treatment escalation.”

## Data Availability

The datasets presented in this study can be found in online repositories. The names of the repository/repositories and accession number(s) can be found in the article/supplementary material.
